# Antibacterial Potential of *Caesalpinia coriaria* (Jacq) Willd Fruit against *Aeromonas* spp. of Aquaculture Importance

**DOI:** 10.3390/ani12040511

**Published:** 2022-02-18

**Authors:** Lenin Rangel-López, Nallely Rivero-Perez, Benjamín Valladares-Carranza, Agustín Olmedo-Juárez, Lucía Delgadillo-Ruiz, Vicente Vega-Sánchez, Sawako Hori-Oshima, Mohamed A. Nassan, Gaber El-Saber Batiha, Adrian Zaragoza-Bastida

**Affiliations:** 1 Instituto de Ciencias Agropecuarias, Área Académica de Medicina Veterinaria y Zootecnia, Universidad Autónoma del Estado de Hidalgo, Rancho Universitario Av. Universidad km 1, Ex-Hda. de Aquetzalpa, Tulancingo C.P. 43600, Hidalgo, Mexico; ra415670@uaeh.edu.mx (L.R.-L.); vicente_vega11156@uaeh.edu.mx (V.V.-S.); 2 División Académica en Ciencias Agropecuarias, Universidad Juárez Autónoma de Tabasco, Carretera Villahermosa-Teapa Kilómetro 25+2 Ranchería la Huasteca 2da sección, Villahermosa C.P. 86298, Tabasco, Mexico; 3 Centro de Investigación y Estudios Avanzados en Salud Animal, Facultad de Medicina Veterinaria y Zootecnia, Universidad Autónoma del Estado de México, km 15.5 Carretera Panamericana Toluca-Atlacomulco, Toluca C.P. 50200, Estado de México, Mexico; bvalladaresc@uaemex.mx; 4 Centro Nacional de Investigación Disciplinaria en Salud Animal e Inocuidad (CENID SAI-INIFAP), Carretera Federal Cuernavaca-Cuautla No. 8534/Col. Progreso, Jiutepec C.P. 62550, Morelos, Mexico; olmedo.agustin@inifap.gob.mx; 5 Unidad Académica de Ciencias Biológicas, Universidad Autónoma de Zacatecas, Zacatecas C.P. 98000, Zacatecas, Mexico; luciadelgadillo@uaz.edu.mx; 6 Instituto de Investigaciones en Ciencias Veterinarias, Universidad Autónoma de Baja California, Mexicali C.P. 21000, Baja California, Mexico; shori@uabc.edu.mx; 7 Department of Clinical Laboratory Sciences, Turabah University College, Taif University, P.O. Box 11099, Taif 21944, Saudi Arabia; m.nassan@tu.edu.sa; 8 Department of Pharmacology and Therapeutics, Faculty of Veterinary Medicine, Damanhour University, Damanhour 22511, Egypt; dr_gaber_batiha@vetmed.dmu.edu.eg

**Keywords:** antiaeromonas activity, hydroalcoholic extract, *Caesalpinia coriaria* fruit, *Aeromonas hydrophila*, *A. dhakensis*, *A. veronii*, *Oncorhybchus mykiss*, *Oreochromis* spp.

## Abstract

**Simple Summary:**

Aquaculture remains an important source of food, however, aquaculture systems are affected by different factors including the appearance of resistant or multiresistant bacteria to antimicrobials. An alternative in the search for new treatments for these bacteria is plant extracts. The aim of the present study was to determine the antibacterial activity of *Caesalpinia coriaria* fruit hydroalcoholic extract and gallic acid over *Aeromonas hydrophila*, *Aeromonas veronii*, and *Aeromonas dhakensis* to identify new molecules for the treatment of diseases caused by *Aeromona* spp. The hydroalcoholic extract of *Caesalpinia coriaria* and its fractions have antibacterial activity against *Aeromonas hydrophila*, *Aeromonas veronii,* and *Aeromonas dhakensis* and could be alternatives for the treatment of diseases caused by the genus *Aeromonas*.

**Abstract:**

Aquaculture is an important source of food and livelihood for hundreds of millions of people around the world, however, aquaculture systems are affected by different factors, among them the appearance of resistant or multiresistant bacteria to antimicrobials. The secondary metabolites of plants have been proposed as alternatives for the treatment of these bacteria. The aim of the present study was to determine the antibacterial activity of *Caesalpinia coriaria* fruit hydroalcoholic extract and gallic acid over *Aeromonas hydrophila*, *Aeromonas veronii,*  and *Aeromonas dhakensis* to identify new molecules for the treatment of diseases caused by *Aeromonas* spp. The *C. coriaria* fruit hydroalcoholic extract (HECc) was obtained by hydroalcoholic maceration and subjected to bipartition with ethyl acetate and water to obtain an aqueous fraction (Ac-FrCc) and an organic fraction (Ac-FrEtCc); gallic acid was purchased commercially. The Minimum Inhibitory Concentration (MIC), Minimum Bactericidal Concentration (MBC), MBC/MIC ratio, and cytotoxicity of HECc, its fractions, and gallic acid were determined. The results indicate that HECc fractions (Ac-FrCc and Ac-FrEtCc) and gallic acid have bactericidal activity against *A. hydrophila* and *A. dhakensis,* but only gallic acid showed bactericidal activity against *A. veronii*. The HECc and Ac-FrCc showed no toxicity, Ac-FrEtCc showed low toxicity, and gallic acid showed medium toxicity. The HECc, Ac-FrCc, and Ac-FrEtCc may be alternatives for the treatment of diseases caused by the genus *Aeromonas*, however, in vivo assays are necessary to corroborate these results.

## 1. Introduction

Aquaculture is an agricultural activity that generates 82 million tons of fish and seafood worldwide [1]. In 2018, Mexico produced 395,000 tons of aquaculture products, the main species cultivated being tilapia and trout [2].

Currently, aquaculture has achieved fish cultures in high density, a situation that makes aquatic organisms more sensitive to pathogens [3], such as bacteria, which limit the production [4], causing serious health problems associated with the low expression of productive parameters in aquatic organisms and economic losses to producers [5].

To prevent or treat diseases of bacterial origin in aquaculture, antimicrobials from veterinary medicine are used. These have been incorrectly used as feed additives and growth promoters in some production systems [6]. The most used antimicrobials in aquaculture are enrofloxacin, oxytetracycline, and florfenicol [7]; the latter was authorized by the FDA (U.S. Food and Drug Administration) in 2005 for use in aquaculture [8].

Rainbow trout (*Oncorhynchus mykiss*) and tilapia (*Oreochromis* spp.) cultures are affected by several bacterial genera, including *Aeromonas*, which cause high mortality rates [9]; this genus comprises a group of Gram-negative bacteria that grow at temperatures of 22–37 °C, are facultative anaerobes, and can live in brackish and freshwater environments [10].

In rainbow trout, *Aeromonas hydrophila* generates abscesses and ulcers in the kidney, spleen, and liver and hemorrhages in the gills and anus [11]. *A. veronii* affects *Oreochromis* sp. causing ulcers with muscle necrosis, hemorrhages on the body surface and in the base of the fins, anal prolapse, and lesions in the bladder, kidneys, liver, spleen, gall bladder, heart, brain, and intestine [12]. *A. dhakensis*, considered a subspecies of *A. hydrophila* [13], affects tilapia, in which it causes hemorrhages from the operculum to the pectoral fins, erosions in caudal fins, abdominal distention, anal prolapse, lethargy, and anorexia [14].

The resistance of *Aeromonas* species to different antimicrobials has been reported: *A. hydrophila* has presented resistance to penicillin, amoxicillin, piperacillin, cephalexin, doxycycline, and teicoplanin [15], while *A. veronii* has shown resistance to chloramphenicol, enrofloxacin, and kanamycin [16]; the resistance of *A. dhakensis* to erythromycin, amoxicillin, and ampicillin has also been reported [14].

To reduce the use of antimicrobials and to have effective treatments, functional, innocuous, and environmentally friendly alternatives have been sought. Plant extracts, due to their secondary metabolite content [17], have been proposed as some of these alternatives since these metabolites confer diverse biological activities, including antibacterial activity [18].

In 2020, Rangel-López et al. [19], determined the antibacterial activity of the hydroalcoholic extract of *Salix babylonica* over *A. hydrophila*, reporting a Minimum Inhibitory Concentration (MIC) of 25 mg/mL and a Minimum Bactericidal Concentration (MBC) of 100 mg/mL. Lee et al. [20], demonstrated the antibacterial activity of the methanolic extract of *Peperomia pellucida* against *A. hydrophila* isolated from a red tilapia hybrid (*Oreochromis* sp.), determining a MIC of 31.5 mg/mL. In this regard, Taynapun et al. [21] evaluated the ethanolic and aqueous extract of *Caesalpinia sappan* on *A*. *veronii*, obtaining a MIC of 0.469 mg/mL.

*Caesalpinia coriaria* is a tree native to tropical America and the West Indies, known as cascalote in Mexico; there are reports of its potential biological activities, including anti-inflammatory, analgesic, antidiarrheal, antiarthritic, anti-acne, hepatoprotective, anticancer, and antimicrobial [22]. The leaves and fruit of *C. coriaria* have been reported to contain saponins, tannins, flavonoids, ethyl gallate and gallic acid [23,24].

Regarding the antibacterial activity of *C. coriaria*, Rojas et al. [25] evaluated the antibacterial activity of different plants, including *C. coriaria*, determining activity on *Staphylococcus aureus* (20 mg/mL), *Enterococcus faecalis* (290 mg/mL), and *Pseudomonas aeruginosa* (270 mg/mL). Jeeva et al. [26] used *C. coriaria* leaves to make extracts and evaluate their antibacterial activity, observing a positive effect at 10 mg/mL on *Escherichia coli* (6.66 mm), *P. aeruginosa* (13.6 mm), *Klebsiella pneumoniae* (10.0 mm), and *S. aureus* (6.66 mm). Cruz [27] evaluated 0.5 g of dried fruit of *C. coriaria* mixed with sodium thioglycolate on *E. coli*, *P. aeruginosa*, *K. pneumoniae,* and *Streptococcus* *pyogenes*, determining that the concentration was effective against all the bacteria evaluated except for *E*. *coli*.

Olmedo-Juárez et al. [22] evaluated the *C. coriaria* fruit hydroalcoholic extract, the aqueous and organic fractions, as well as the compounds methyl gallate and gallic acid isolated from the organic fraction, on *E. coli*, *P. aeruginosa*, *S. typhi*, *Listeria monocytogenes,* and *S. aureus*, determining that the extract and fractions presented antibacterial activity against the bacteria evaluated and also reporting that the gallic acid identified as the major compound in the organic fraction presented the best MIC against *S. typhi* (0.15 mg/mL) and the best MBC against *P. aeruginosa* and *L. monocytogenes* (5.0 mg/mL).

Therefore, the aim of the present study was to determine the antibacterial activity of *Caesalpinia coriaria* fruit hydroalcoholic extract and gallic acid over *Aeromonas hydrophila*, *Aeromonas veronii,* and *Aeromonas dhakensis* to identify new molecules for the treatment of diseases caused by *Aeromona* spp.

## 2. Materials and Methods

### 2.1. Plant Material

*Caesalpinia coriaria* fruit was collected in Palmar Grande locality, Amatepec municipality, Mexico State (18°23′24.8″ N, 100°17′03.5″ W), with identification voucher 35274.

### 2.2. Hydroalcoholic Extract

The hydroalcoholic extract of *C. coriaria* (HECc) was obtained according to the methodology described by Olmedo-Juárez et al. [22]. A total of 1000 g of *C. coriaria* fruit were macerated in a hydroalcoholic solution (30% methanol/70% water) for 48 h at room temperature. The extract was filtered and concentrated in a rotary evaporator (Büchi R-300, Flawil, Switzerland).

### 2.3. Hydroalcoholic Extract Bipartition

The HECc (60 g) was subjected to liquid-liquid chromatographic separation with water and ethyl acetate (Merck, Darmstadt, Germany) in a funnel to obtain two fractions, an ethyl acetate fraction (Ac-FrEtCc) and an aqueous fraction (AcFr-Cc). The solvents were removed in a rotary evaporator [24].

### 2.4. Gallic Acid

Gallic acid of 99.9% purity was acquired commercially (Sigma-Aldrich, G7384, St. Louis, MO, USA).

### 2.5. Bacteria and Culture Conditions

The strains used were *A. hydrophila* CAIM^347^ (isolated from rainbow trout mouth lesions), *A. veronii* CAIM^1877^ (isolated from tilapia brain), and *A. dhakensis* CAIM^1873^ (isolated from tilapia eye lesions), which were obtained from the Collection of Microorganisms of Aquatic Importance (CAIM) from the Mazatlan Center for Food Research and Development, Sinaloa, Mexico. Strains were reactivated according to the methodology described by Rangel-López et al. [19].

### 2.6. Antimicrobial Sensitivity Testing

Antimicrobial sensitivity was determined following the methodology described in CLSI guidelines [28]. The antimicrobials used were cephalotin, cefotaxime, ciprofloxacin, chloramphenicol, nitrofurantoin, ampicillin, carbenicillin, gentamicin, netilmicin, norfloxacin, sulfamethoxazole/trimethoprim, and amikacin.

### 2.7. Antibacterial Activity

To evaluate the antibacterial potential, the MIC (Minimum Inhibitory Concentration) and MBC (Minimum Bactericidal Concentration) of HECc, Ac-FrEtCc, Ac-FrCc, and gallic acid were determined according to the methodology described by Olmedo-Juárez et al. [22]. MIC was determined using the microdilution plate method by colorimetry based on the use of tetrazolium salts [29]; for HECc, the concentrations evaluated were from 200 to 1.56 mg/mL, for Ac-FrEtCc, Ac-FrCc, and gallic acid, the concentrations evaluated were from 6.25 to 0.04 mg/mL; each treatment was evaluated in triplicate.

In a 96-well plate, 100 µL of each of the concentrations of HECc, Ac-FrEtCc, Ac-FrCc, gallic acid and 10 µL of a bacterial suspension adjusted to 0.5 McFarland’s (Remel, R20421, Lenexa, KS, USA) were added. The plate was incubated at 30 °C for 24 h. Afterwords, 20 µL of a solution of p-iodonitrotetrazolium (0.04%, *w*/*v*) (Sigma-Aldrich, 18377, St. Louis, MO, USA) was added to each well, followed by incubation for 30 min at 30 °C. The concentration at which the solution turned pink was determined as the MIC [30].

Prior to the addition of p-iodonitrotetrazolium, 5 µL of each well was taken, inoculated on Mueller-Hinton agar (DIBICO^®^, Mexico City, Mexico), and incubated at 30 °C for 24 h. The lowest concentration of each treatment at which no bacterial growth was observed was determined as MBC [31].

### 2.8. Bactericidal and Bacteriostatic Effect

To determine the bactericidal or bacteriostatic effect of HECc, Ac-FrEtCc, Ac-FrCc, and gallic acid, the MBC/MIC ratio was calculated, considering that any value of ≤4 indicated a bactericidal effect and that a value of >4 was indicative of a bacteriostatic effect [32].

### 2.9. Cytotoxicity Test with Artemia salina

The cytotoxicity of HECc, Ac-FrEtCc, Ac-FrCc, and gallic acid were determined by the microdilution plate assay with *Artemia salina* according to the methodology described by Solis et al. [33] and Rivero-Pérez et al. [34], with some modifications. Cysts of *Artemia salina* were hatched in saline solution (38 g/L) for 24 h at 25 °C. Subsequently, in a 96-well plate, serial dilutions were performed to obtain concentrations of 37–0.090 mg/mL in 100 µL of saline; after, 100 µL of saline solution containing between 10 and 15 nauplii of *Artemia salina* were added and count verified in a stereoscopic microscope (Eco SZ-745, Schertz, TX, USA). Tween^®^80 (SIGMA P1754, St. Louis, MO, USA) was used as a positive control and saline as a negative. Once the plate was ready, it was incubated at 25 °C for 24 h. After which, each well was observed under a stereoscopic microscope to count dead and live nauplii and determine the mortality percentage using the formula proposed by Sulit and Atienza [35].

(1) $$\mathrm{Mortality}\;=\frac{\mathrm{Number}\;\mathrm{of}\;\mathrm{dead}\;\mathrm{nauplii}}{\mathrm{Initial}\;\mathrm{number}\;\mathrm{of}\;\mathrm{nauplii}}\times 100$$

To determine the degree of toxicity of HECc, Ac-FrEtCc, Ac-FrCc, and gallic acid, the criteria described by Mentor et al. [36] were used as a reference to the Lethal Dose 50 (LD_50_); this established that values >1.0 mg/mL are indicative that the product is not toxic, values of 0.5–1.0 mg/mL indicate low toxicity, 0.1–0.5 mg/mL indicate medium toxicity, and values less than 0.1 mg/mL are indicative of high toxicity.

### 2.10. Statistical Analysis

The MIC and MBC data were normalized and analyzed by analysis of variance (ANOVA) and Tukey’s comparison of means (*p* < 0.05). The LD_50_ values of the extract, fractions, and gallic acid were determined by *Probit* analysis. Statistical analyses were performed in SAS software version 9.0 (SAS Institute, Cary, NC, USA).

## 3. Results

### 3.1. Antimicrobial Sensitivity

The measurement of inhibition halos to determine antimicrobial sensitivity indicated that *A. hydrophila* was resistant to cephalothin (14 mm), ampicillin (6 mm), and carbenicillin (6 mm). *A. veronii* was resistant to ampicillin (6 mm), carbenicillin (6 mm), and amikacin (6 mm). *A. dhakensis* was resistant to cephalothin (8 mm), ampicillin (6 mm), carbenicillin (6 mm), and amikacin (6 mm); the results are shown in Table 1.

### 3.2. Antibacterial Activity

The results of the MIC of HECc, Ac-FrCc, Ac-FrEtCc, and gallic acid indicated their capacity to inhibit the growth of the strains evaluated to different concentrations (*p* = 0.0001). HECc showed a higher potential toward *A. veronii* and *A. dhakensis* (0.78 mg/mL) and lower activity toward *A. hydrophila* (1.56 mg/mL); the best MIC of Ac-FrCc was obtained against *A. hydrophila* (0.19 mg/mL), followed by *A. veronii* (0.39 mg/mL) and *A. dhakensis* (0.39 mg/mL). With respect to Ac-FrEtCc, the highest MIC was against *A. hydrophila* (0.09 mg/mL), followed by *A. veronii* and *A. dhakensis* (0.78 mg/mL). For gallic acid, the best MIC was against *A. hydrophila* (0.09 mg/mL), followed by *A. veronii* (3.12 mg/mL) and without activity on *A. dhakensis* (Table 2).

Analyzing the treatment effects on the growth inhibition of each bacterium, significant statistical differences were observed (*p* = 0.0001). For *A. hydrophila*, Ac-FrEtCc and gallic acid (0.09 mg/mL) presented better inhibitory activity, followed by Ac-FrCc (0.19 mg/mL) with the lowest activity shown by HECc (1.56 mg/mL). For *A. veronii,* Ac-FrCc (0.39 mg/mL) had the highest activity, followed by HECc and Ac-FrEtCc (0.78 mg/mL); the lowest activity being observed with gallic acid (3.12 mg/mL). For *A. dhakensis*, the highest activity was obtained with Ac-FrCc (0.39 mg/mL), followed by HECc and Ac-FrEtCc (0.78 mg/mL), while gallic acid showed no activity over this species (Table 2).

When analyzing the MBCs of HECc, Ac-FrEtCc, Ac-FrCc, and gallic acid on the strains evaluated, significant statistical differences (*p* = 0.0001) were determined. For HECc, the best MBC was presented toward *A. dhakensis* (1.56 mg/mL), followed by *A*. *hydrophila* (3.12 mg/mL) and *A. veronii* (6.25 mg/mL). For Ac-FrCc the best effect was observed against *A. hydrophila* (0.19 mg/mL), followed by *A. dhakensis* (0.78 mg/mL) and finally *A. veronii* (3.12 mg/mL). Ac-FrEtCc showed the highest activity on *A. hydrophila* (0.19 mg/mL), followed by *A. dhakensis* (3.12 mg/mL) and *A. veronii* (6.25 mg/mL). Gallic acid was better at killing *A. hydrophila* (0.09 mg/mL), followed by *A. veronii* (6.25 mg/mL), with no activity on *A. dhakensis*.

Regarding the bacteria evaluated, significant statistical differences (*p* = 0.0001) were observed when comparing the bactericidal activity of the treatments evaluated. Against *A. hydrophila*, gallic acid (0.09 mg/mL) presented the best MBC, followed by Ac-FrEtCc and Ac-FrCc (0.19 mg/mL) with less activity shown by HECc (3.12 mg/mL). With respect to *A. veronii*, Ac-FrCc (3.12 mg/mL) was the most active, followed by HECc, Ac-FrEtCc, and gallic acid (6.25 mg/mL). For *A. dhakensis*, Ac-FrCc (0.78 mg/mL) presented the highest bactericidal activity, followed by HECc (1.56 mg/mL) and Ac-FrEtCc (3.12 mg/mL); the gallic acid did not present bactericidal activity (Table 3).

### 3.3. Bacteriostatic and Bactericidal Activity

The results obtained for the MBC/MIC ratio of HECc, Ac-FrEtCc, Ac-FrCc, and gallic acid determined that the activities against *A. hydrophila* were bactericidal. The effects against *A. veronii* of HECc, Ac-FrEtCc, and Ac-FrCc were bacteriostatic but bactericidal for gallic acid. On *A. dhakensis*, HECc, Fr-EtAcCc, and Ac-FrCc showed a bactericidal effect, while gallic acid showed no activity (Table 4).

### 3.4. Cytotoxicity of Extract, Fractions, and Gallic Acid against Artemia salina

**Figure 1:**
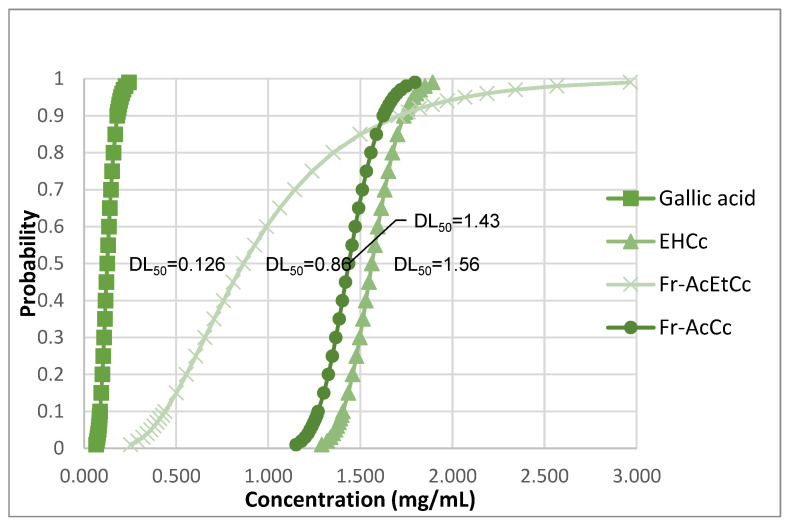
LD_50_ of the hydroalcoholic extract and fractions of the *Caesalpinia coriaria* fruit and gallic acid.

Results obtained for the LD_50_ are shown in Figure 1 and, according to the criteria used for result interpretation, HECc (1.56 mg/mL) and Ac-FrCc (1.43 mg/mL) were not toxic, Ac-FrEtCc (0.86 mg/mL) showed low toxicity, and gallic acid (0.126 mg/mL) showed medium toxicity.

## 4. Discussion

In aquaculture, several strategies have been used with the objective to increase stocking densities in production units; some of which affect fish health negatively, generating stress and sensitivity to bacterial diseases such as those caused by the genus *Aeromonas* spp. And whose treatment is based on the use of antimicrobials that has led to the emergence of resistant or multiresistant-drug strains [37].

In the present study, antimicrobial sensitivity testing was performed on *A. hydrophila*, *A. veronii,* and *A. dhakensis*. The results indicated that *A. hydrophila* was resistant to beta-lactams (ampicillin and carbenicillin) and first-generation cephalosporins (cephalothin). In this regard, Stratev and Odeyemi [38] reported the resistance of *A. hydrophila* to cephalothin, ampicillin, and carbenicillin, coincident with our results. On the other hand, *A*. *veronii* was resistant to beta-lactams (ampicillin and carbenicillin) and aminoglycosides (amikacin). Yu et al. [39] determined that *A. veronii* presented resistance to aminoglycosides (kanamycin), tetracyclines (oxytetracycline), and beta-lactams (ampicillin), partially confirming the results of the present study, while *A. dhakensis* presented resistance to ampicillin, carbenicillin, amikacin, and cephalothin. In the same sense, Soto-Rodríguez et al. [14] reported that *A. dhakensis* was resistant to macrolides and beta-lactams, coinciding, for this latter group, with the present study (Table 1).

In accordance with the above and with the criteria described by López-Pueyo et al. [40], it is considered that the *A. hydophila* and *A. dhakensis* strains evaluated in this study are multidrug-resistant to antimicrobials since they presented resistance to more than one family of antimicrobials (beta-lactams and aminoglycosides), in addition to being of clinical and epidemiological relevance.

In the evaluation of antibacterial activity, HECc presented better activity, inhibiting the growth of *A. veronii* and *A. dhakensis* (0.78 mg/mL), while on *A. hydrophila,* the effect was observed at 1.56 mg/mL (Table 2). Bandeira et al. [5] evaluated 10 essential oils and determined a MIC of 1.6 mg/mL toward *A. dhakensis* using *Illicium verum* essential oil. Hardi et al. [41] determined growth inhibition of *A. hydrophila* by an ethanolic extract of *Curcuma aeruginosa* at 500 mg/mL (10 mm halo) while Kavitha et al. [42] observed an inhibition halo of 14 mm on *A. veronii* with ethyl acetate extract of *Azadirachta indica* at 0.025 mg/mL. Pachanawan et al. [43] reported that the aqueous extract of *Andrographis paniculata* at 0.5 mg/mL produced a halo of 14.6 mm on *A. hydrophila*. However, these results are not fully comparable since a different plant source, different solvents, and a different methodology was used to determine the antibacterial activity.

When determining the MIC of the two fractions obtained from the hydroalcoholic extract of *C. coriaria*, it was observed that Ac-FrEtCc was more active against *A*. *hydrophila* (0.09 mg/mL) and that Ac-FrCc showed a greater activity against *A. veronii* and *A. dhakensis* (0.39 mg/mL for both) (Table 2). However, so far there is no information regarding the determination of the MIC toward bacteria of aquaculture importance of fractions obtained from plant extracts; although in 2019, Olmedo-Juárez and collaborators [22] determined that the organic fraction of *C. coriaria* fruit was more active in inhibiting the growth of *E. coli*, *P. aeruginosa*, *S. typhi*, *L. monocytogenes*, and *S. aureus* than was the aqueous fraction.

Gallic acid inhibited the growth of *A. hydrophila* (0.09 mg/mL) and *A. veronii* (3.12 mg/mL) but showed no activity against *A. dhakensis* (Table 2). Prasad et al. [44] determined a MIC of 0.96 mg/mL for gallic acid against *A. hydrophila*, a value higher than that reported in this study, while Chug et al. [45] found no inhibition of this bacterium at 5 mg/mL by gallic acid diluted in deionized water, a result contrary to that reported in the present study. In addition, Olmedo-Juárez et al. [22] determined that gallic acid also presented inhibitory activity on Gram-positive and Gram-negative bacteria affecting public health at concentrations of 0.15–2.50 mg/mL, similar concentrations to those reported in the present experiment.

With respect to MBC, it was determined that HECc presented activity over *A. hydrophila*, *A. veronii,* and *A. dhakensis* (3.14, 6.25, and 1.56 mg/mL, respectively) (Table 3). Kanchan et al. [46] evaluated the ethanolic extract of *Terminalia catappa* against *A*. *hydrophila* and reported bactericidal activity at 12.5 mg/mL. Olmedo-Juárez et al. [22] evaluated HECc on bacteria of public health importance and observed MBC values of 25–100 mg/mL, concentrations higher than those reported in the present study.

When determining the MBC of the fractions, higher activity was observed for both Ac-FrEtCc and Ac-FrCc against *A. hydrophila* (0.19 mg/mL), followed by Ac-FrCc over *A*. *dhakensis* (0.78 mg/mL) (Table 3). In this sense, Olmedo-Juárez et al. [22] determined that the organic fraction at concentrations of 6.2–25 mg/mL and the aqueous fraction at concentrations of 25–100 mg/mL presented bactericidal activity against *E. coli*, *P. aeruginosa*, *S. typhi*, *L. monocytogenes,* and *S. aureus*.

In the case of gallic acid, the best MBC was obtained against *A. hydrophila* (0.09 mg/mL), followed by *A. veronii* (6.25 mg/mL), with no activity against *A. dhakensis* (Table 3). It is worth mentioning that there are no studies in which the MBC of gallic acid toward these bacteria has been determined. Olmedo-Juárez et al. [22] determined that gallic acid also showed activity against bacteria of public health importance at concentrations of 5–10 mg/mL.

According to reports by Soberón et al. [47] and Rivas-Cáceres et al. [48], it is more relevant to identify compounds with bactericidal rather than bacteriostatic activity since extracts are sought that eliminate the bacteria rather than just inhibit their growth. In the present experiments, the results obtained for the MBC/MIC ratio indicate that HECc, Ac-FrEtCc, and Ac-FrCc have bactericidal activity against *A*. *hydrophila* and *A. dhakensis*, while gallic acid has bactericidal activity against only *A*. *hydrophila* and *A*. *veronii*, presenting no activity toward *A. dhakensis* (Table 4). Rangel-López et al. [19] reported that the hydroalcoholic extract of *Salix babylonica* has bactericidal activity against *A*. *hydrophila* (100 mg/mL), both results coinciding with those reported in the present study.

Regarding the mechanism of action, Mohana and Raveesha [23] reported that the antibacterial activity of the methanolic extract of *C. coriaria* can be associated with the presence of phenolic compounds and acids; however, they are not the only secondary metabolites present in *C. coriaria*. Pizzanin et al. [49] reported the presence of phenols, condensed tannins, proanthocyanidins, flavonoids, tannins, quinones, coumarins, and saponins. Olmedo-Juárez et al. [22] reported that the antibacterial activity of *C. coriaria* fruit is associated with the presence of methyl gallate and gallic acid in its organic fraction and that these compounds can cause hyperpolarization of the cell membrane and thus destabilize and alter the permeability of the bacterial cell surface, thus causing its destruction.

According to the criteria described by Mentor et al. [37] and Rivero-Perez et al. [35], HECc (LD_50_ 1.56 mg/mL) and Ac-FrCc (LD_50_ 1.43 mg/mL) do not present toxicity (Figure 1); similar results were reported by Ávalos-Soto et al. [50] who determined an LD_50_-value of >1 mg/mL for the ethanolic extract of the *Azadirachta indica* seed husk. Ac-FrEtCc (LD_50_ 0.86 mg/mL) presented low toxicity and gallic acid medium toxicity (LD_50_ 0.126 mg/mL) (Figure 1); this may be associated with the purification of fractions with higher biological activity, as reported by Apu et al. [51].

## 5. Conclusions

The results of this study indicate that the hydroalcoholic extract obtained from the fruit of *C. coriaria* contains secondary metabolites with antibacterial potential against *A. hydrophila*, *A. veronii,* and *A. dhakensis* since it was observed that the extract, its two fractions, and gallic acid inhibit their growth and at certain concentrations even kill them. The results indicate a bactericidal activity over *A. hydrophila* and *A. dhakensis*, activity highly desirable in limiting the emergence of bacterial strains resistant to the compounds present in the extract and its fractions. The cytotoxicity tests performed with HECc, Ac-FrCc, and Ac-FrEtCc indicated that they have low toxicity which guarantees its effectiveness and safety for later studies in in vivo models in which the antiaeromonas potential of the *C. coriaria* fruit and its possible route of administration can be evaluated.

## Figures and Tables

**Table 1:** Results of inhibition halos (mm) and antibiotic sensitivity of *A. hydrophila*, *A. veronii,* and *A. dhakensis*.

Antibiotic	*A. hydrophila*	*A. veronii*	*A. dhakensis*
Cephalotin (30 µg)	14(R)	25(S)	8(R)
Cefotaxime (30 µg)	30(S)	30(S)	33(S)
Ciprofloxacin (5 µg)	25(S)	30(S)	25(S)
Cloramphenicol (30 µg)	25(S)	30(S)	30(S)
Nitrofurantoin (300 µg)	25(S)	22(S)	25(S)
Ampicillin (10 µg)	6(R)	6(R)	6(R)
Carbenicillina (100 µg)	6(R)	6(R)	6(R)
Gentamicin (10 µg)	20(S)	15(S)	20(S)
Netelmicin (30 µg)	20(S)	10(I)	10(I)
Norfloxacin (10 µg)	20(S)	15(I)	15(I)
Sulfamethoxazole/Trimethoprim (25 µg)	22(S)	12(S)	20(S)
Amikacin (30 µg)	20(S)	6(R)	6(R)

S: sensitive, R: resistant, I: intermediate.

**Table 2:** MIC of HECc, Ac-FrCc, Ac-FrEtCc, and gallic acid, against *A. hydrophila*, *A. veronii*, and *A. dhakensis*.

Treatment	Minimal Inhibitory Concentration (mg/mL)		
	*A. hydrophila*	*A. veronii*	*A. dhakensis*
EHCc	1.56 ^cB^	0.78 ^bA^	0.78 ^bA^
Ac-FrEtCc	0.09 ^aA^	0.78 ^bB^	0.78 ^bB^
Ac-FrCc	0.19 ^bA^	0.39 ^aB^	0.39 ^aC^
Gallic acid	0.09 ^aA^	3.12 ^cB^	NA
P.C. (Kanamycin µg/mL)	1 ^A^	4 ^C^	2 ^B^
N.C.	NA	NA	NA
Valor de *p*	0.0001	0.0001	0.0001

HECc: *C. coriaria* hydroalcoholic extract, Ac-FrEtCc: ethyl acetate fraction, Ac-FrCc: aqueous fraction, P.C: positive control, N.C: negative control, NA: no activity, ^a,b,c^ different literals in the columns indicate significant statistical differences (*p* ≤ 0.05), ^A,B,C^ different literals in the row indicate significant statistical differences (*p* ≤ 0.05).

**Table 3:** MBC of HECc, Ac-FrCc, Ac-FrEtCc, and gallic acid against *A. hydrophila*, *A. veronii* y *A. dhakensis*.

Treatment	Minimal Bactericidal Concentration (mg/mL)		
	*A. hydrophila*	*A. veronii*	*A. dhakensis*
HECc	3.12 ^cB^	6.25 ^bC^	1.56 ^bA^
Ac-FrEtCc	0.19 ^bA^	6.25 ^bC^	3.12 ^cB^
Ac-FrCc	0.19 ^bA^	3.12 ^aA^	0.78 ^cB^
Gallic acid	0.09 ^aA^	6.25 ^bB^	NA
P.C. (µg/mL)	2 ^B^	16 ^C^	1 ^A^
N.C.	NA	NA	NA
Valor de *p*	0.0001	0.0001	0.0001

HECc: *C. coriaria* fruit hydroalcoholic extract, Ac-FrEtCc: ethyl acetate fraction, Ac-FrCc: aqueous fraction, P.C: positive control, N.C: negative control, NA: no activity, ^a,b,c^ different literals in the columns indicate significant statistical differences (*p* ≤ 0.05), ^A,B,C^ different literals in the row indicate significant statistical differences (*p* ≤ 0.05).

**Table 4:** MBC/MIC ratio of the hydroalcoholic extract, fractions, and gallic acid.

Treatment	Ratio of MBC/MIC		
	*A. hydrophila*	*A. veronii*	*A. dhakensis*
HECc	2.0 (Bactericidal)	8.0 (Bacteriostatic)	2.0 (Bactericidal)
Ac-FrEtCc	2.1 (Bactericidal)	8.0 (Bacteriostatic)	4.0 (Bactericidal)
Ac-FrCc	1.0 (Bactericidal)	8.9 (Bacteriostatic)	2.0 (Bactericidal)
Gallic acid	1.0 (Bactericidal)	2.0 (Bactericidal)	NA

HECc: *C. coriaria* fruit hydroalcoholic extract, Ac-FrEtCc: ethyl acetate fraction, Ac-FrCc: aqueous fraction, MBC: Minimal Bactericidal Concentration, MIC: Minimum Inhibitory Concentration, NA: No Activity.

## Data Availability

Data are contained within the article.

## References

[1] FAO (2020). El Estado Mundial de la Pesca y La Acuicultura 2020. La Sostenibilidad en Acción.

[2] CONAPESCA (2018). Anuario Estadístico de Acuacultura y Pesca. https://www.conapesca.gob.mx/work/sites/cona/dgppe/2018/ANUARIO_2018.pdf.

[3] Bao L., Chen Y., Li H., Zhang J., Wu P., Ye K., Ai H., Chu W. (2019). Dietary Ginkgo biloba leaf extract alters immune-related gene expression and disease resistance to Aeromonas hydrophila in common carp Cyprinus carpio. Fish Shellfish Immunol..

[4] Abdel-Latif H.M.R., Dawood M.A.O., Menanteau-Ledouble S., El-Matbouli M. (2020). The nature and consequences of co-infections in tilapia: A review. J. Fish Dis..

[5] Bandeira J.G., Souza C.F., Dellaméa B.M., Nunes D.S., Petri S.B., Tasca C., Veras M.R.H., Palmira C.V.A., Baldisserotto B. (2019). Plant essential oils against bacteria isolated from fish: An in vitro screening and in vivo efficacy of Lippia origanoides. Cienc. Rural..

[6] Reverter M., Botemps N., Lecchini D., Banaigs B., Sasal P. (2014). Use of plant extracts in fish aquaculture as an alternative to chemotherapy: Corrent status and future perspectives. Aquaculture.

[7] Garcia-Valenzuela M.H., Orozco-Medina C., Molina-Maldonado C. (2012). Efecto antibacteriano del aceite esencial de orégano (Lipia berlandieri) en bacterias patógenas de camarón Litopenaeus vannamei. Hidrobiológica.

[8] Culot A., Grosset N., Gautier M. (2019). Overcoming the challenges of phage therapy for industrial aquaculture: A review. Aqua-culture.

[9] Starliper C.E., Ketola H.G., Noyes A.D., Schill W.B., Henson F.G., Chalupnicki M.A., Dittman D.E. (2014). An investigation of the bactericidas activity of selected essential oils to *Aeromonas* spp.. J. Adv. Res..

[10] Martin-Carnahan A., Joseph S.W. (2015). Aeromonas. Bergey’s Manual of Systematics of Archaea and Bacteria.

[11] Constantino C.F., Armijo O.A., Osorio S.D., Chávez S.L. (1997). Infección por Aeromonas hydrophila e Ichthyophthirius multifiliis en trucha (Oncorhynchus mykiss, Walbaum) y tilapia (Oreochromis aureus, L) de un centro de acopio de Morelos, México. Estudio patológico. Vet. Mex..

[12] Eissa I.A.M., El-Lamei M., Sherif M., Desuky E., Zaki M., Bakry M. (2015). Aeromonas veronii biovar sobria a causative agent of mass mortalities in cultured Nile tilapia in El-Sharkia governorate. Egypt. Life Sci. J..

[13] Esteve C., Alcaide E., Blasco M.D. (2012). Aeromonas hydrophila subsp. dhakensis isolated from feces, water and fish in mediterranean spain. Microbes Environ..

[14] Soto-Rodríguez S.A., Lozano-Olvera R., García-Gasca M.T., Abad-Rosales S.M., Gómez-Gil B., Ayala-Arellano J. (2018). Virulence of the fish pathogen Aeromonas dhakensis genes involved, characterization and histopathology of experimentally infected hybrid tilapia. Dis. Aquat. Org..

[15] Zhu W., Zhou S., Chu W. (2019). Comparative proteomic analisis of sensitive and multidrog resistant Aeromonas hydrophila isolated from deseased fish. Microb. Pathog..

[16] Cai S.-H., Wu Z.-H., Jian J.-C., Lu Y.-S., Tang J.-F. (2011). Characterization of pathogenic Aeromonas veronii bv. Veronii associated with ulcerative syndrome from Chinese longsnout catfish (Leiocassis longirostris Günther). Raz. J. Microbiol..

[17] Van Hai N. (2015). The use of medicinal plants as immunostimulants in aquaculture: A review. Aquaculture.

[18] Harikrishnan R., Balasundaram C., Heo M. (2011). Impacto of plant products on innate and adaptive immune system of cultured finfish and shellfish. Aquaculture.

[19] Rangel-López L., Zaragoza-Bastida A., Valladares-Carranza B., Peláez-Acero A., Sosa-Gutiérrez C.G., Hetta H.F., Batiha G.E., Alqahtani A., Rivero-Perez N. (2020). In vitro antibacterial potential of Salix babylonica extract against bacteria that affect *Oncorhynchus mykiss* and *Oreochromis* spp.. Animals.

[20] Lee S.W., Sim K.Y., Wendy W., Zulhisyam A.K. (2016). Peperomia pellucida leaf extract as immunostimulator in controlling motile aeromonad septicemia due to *Aeromonas hydrophila* in red hibrid tilapia, *Oreochromis* spp.. Farm. Vet. World..

[21] Taynapun K.U., Mueangkan N., Chirapongsatonkul N. (2018). Efficacy of herbal extracts to control multi-antibiotics resistant (MAR) Aeromonas veronii isolated from motile Aeromonas septicemia (MAS)-exhibiting Nile tilapia (*Oreochromis niloticus*). Int. J. Agric. Technol..

[22] Olmedo-Juárez A., Briones-Robles T.I., Zaragoza-Bastida A., Zamilpa A., Ojeda-Ramírez D., Mendoza de Gives P., Olivares-Pérez J., Rivero-Perez N. (2019). Antibacterial activity of compounds isolated from Caesalpinia coriaria (Jacq) willd against important bacteria in public health. Microb. Pathog..

[23] Mohana D.C., Raveesha K.A. (2006). Anti-bacterial activity of Caesalpinia coriaria (Jacq.) Willd. against plant pathogenic Xan-thomonas pathovars: An eco-friendly approach. J. Agric. Sci. Technol..

[24] García-Hernández C., Rojo-Rubio R., Olmedo-Juárez A., Zamilpa A., Mendoza de Gives P., Antonio-Romo I.A., Aguilar-Marcelino L., Arece-García J., Tapia-Maruri D. (2019). Galloyl derivatives from Caesalpinia coriaria exhibit in vitro ovicidal activity against cattle gastrointestinal parasitic nematodes. Exp. Parasitol..

[25] Rojas J., Velasco J., Buitrago A., Mender T., Rojas J. (2016). Evaluación de la actividad antimicrobiana de plantas medicinales seleccionadas del jardín botánico del Orinoco, municipio Heres, Estado Bolivar. Rev. Fac. Farm..

[26] Jeeva K., Thiyagarajan M., Elangovan V., Geetha N., Venkatachalam P. (2014). Caesalpinia coriaria leaf extracts mediated biosyn-thesis of metallic silver nanoparticles and their antibacterial activity against clinically isolated pathogens. Ind. Crops Prod..

[27] Cruz M.C. (2007). Pruebas de sensibilidad y resistencia bacteriana frente a diferentes concentraciones de extracto de Caesalpinia coriaria (Guatanamá). Cienc. Soc..

[28] Clinical and Laboratory Standards Institute (2012). Methods for Dilution Antimicrobial Susceptibility Tests for Bacteria That Grow Aerobically (Approved Standard).

[29] Zaragoza-Bastida A., Flores-Aguilar S.C., Aguilar-Castro L.M., Morales-Ubaldo A.L., Valladares-Carranza B., Rangel-López L., Olmedo-Juárez A., Rosenfeld-Miranda C.E.C., Rivero-Pérez N. (2020). Antibacterial and hemolytic activity of Crotalus triseriatus and Crotalus ravus Venom. Animals.

[30] Morales-Ubaldo A., Rivero-Pérez N., Avila-Ramos F., Aquino-Torres E., Prieto-Mendez J., Hetta H.F., El-Saber Batiha G., Zaragoza-Bastida A. (2021). Bactericidal activity of Larrea tridentata hydroalcoholic extract against phytopathogenic bacteria. Agronomy.

[31] González-Alamilla E., Rivas-Jacobo M., Sosa-Gutiérrez C., Delgadillo-Ruiz L., Valladares-Carranza B., Rosenfeld-Miranada C., Zaragoza-Bastida A., Rivero-Pérez N. (2020). Efecto antibacteriano del extracto metanolico de Salix babylonica sobre bacterias de importancia en salud pública. Abanico Vet..

[32] Djihane B., Wafa N., Elkhamssa S., Haro J.P.D., Angeles E.M., Mouhamed M.Z. (2016). Chemical constituents of Helichrysum italicum (Roth) G. Don essential oil and their antimicrobial activity against Gram-positive and Gram-negative bacteria, fila-mentous fungi and Candida albicans. Saudi Pharm. J..

[33] Solis P.N., Wright C.W., Anderson M.M., Gupta M.P., Phillipson D. (1993). A microwell cytotoxicity assay using Artemia salina (Brine shrimp). Planta Med..

[34] Rivero-Perez N., Hernández-Alvarado J.L., Valladares-Carranza B., Delgadillo-Ruiz L., Ojeda-Ramírez D., Gutiérrez C.G.S., Morales-Ubaldo A.L., Vega-Sanchez V., Zaragoza-Bastida A. (2019). *Salix babylonica* L. as a natural anticoccidial alter-native in growing rabbits. Evid.-Based Complement. Altern. Med..

[35] Sulit J.E.B., Atienza L.M. (2020). Cytotoxic effect of the methanolic extract of selected edible weeds on *Artemia salina* nauplii. J. Nutr. Res. Food Sci..

[36] Mentor R.H., Blagica J., Tatjana K.P. (2014). Toxicological evaluation of the plant products using brine shrimp (*Artemia salina* L.) model. Maced. Pharm. Bull..

[37] Bilen S., Elbeshti H.T.A.G. (2019). A new potential therapeutic remedy against Aeromonas hydrophila infection in rainbow trout (Oncorhynchus mykiss) using tetra Cotinus coggygria. J. Fish Dis..

[38] Stratev D., Odeyemi O.A. (2016). Antimicrobial resistance of Aeromonas hydrophila isolated from different food sources: A mini-review. J. Infect. Public Health..

[39] Yu J.H., Han J.J., Kim H.J., Kang S.G., Park S.W. (2010). Fist reporto Aeromonas veronii infection in farmed Israeli carp Cyprinus carpio in Korea. J. Fish Pathol..

[40] López-Pueyo M.J., Barcenilla-Gaite F., Amaya-Villar R., Garnacho-Montero J. (2011). Multirresistencia antibiótica en unidades de críticos. Med. Intensiva..

[41] Hardi E.H., Kusuma I.W., Suwinarti W., Agustina A.I., Nugroho R.A. (2017). Antibacterial activities of some borneo plant extracts against áthogenic bacteria of *Aeromonas hydrophila* and *Pseudomonas* sp.. AACL Bioflux..

[42] Kavitha M., Raja M., Kamaraj C., Balasubramaniam V., Balasubramaniam G., Perumal P. (2017). In vitro antimicrobial activity of Azadirachta indica (Leaves) against fish pathogenic bacteria isolated from naturally infected Dawkinsia filamentosa (Blacjspot barb). Med. Aromat. Plants..

[43] Pachanawan A., Phumkhachorn P., Rattanachaikunsopon P. (2008). Potential of Psidium guajava suplemented fish diets in controlling Aeromonas hydrophila infection in tilapia (Oreochromis niloticus). J. Biosci. Bioeng..

[44] Prasad V.G.N.V., Krishna B.V., Swamy P.L., Rao T.S., Rao G.S. (2014). Antibacterial synergy between quercetin and poly-phenolic acids against bacterial pathogens of fish. Asian Pac. J. Tropl. Dis..

[45] Chung K.T., Zha G., Stevens E., Simco B.A., Wei C.I. (1995). Groeth inhibition of selected aquatic bacteria by taninic acid and related compounds. J. Aquat Anim Health.

[46] Kanchan C., Imjai P., Kanchan N., Panchai K., Hatai K. (2019). Virulence of Aeromonas hydrophila in Siamese fighting fish (Betta splendens) and the bacterium susceptibility to some herbal plants. Iran. J. Fish. Sci..

[47] Soberón J.R., Sgariglia M.A., Maderuelo M.R.D., Andina M.L., Sampietro D.A., Vattuone M.A. (2014). Antibacterial activities of Ligaria cuneifolia and Jodina rhombifolia leaf extracts against phytopathogenic and clinical bacteria. J. Biosci. Bioeng..

[48] Rivas-Cáceres R.R., Stephano-Hornedo L., Lugo J., Vaca R., Del Aguilla P., Yañez-Ocampo G., Mora-Herrera M.E., Camacho D.L.M., Cipriano-Salazar M., Adeniyi A.P. (2018). Bactericidal effect of silver nanoparticles against the propagation of Clavibacter michiganensis infection in Lycopersicon esculentum Mill. Microb. Pathog..

[49] Pizzani P., Matute I., De Martino G., Arias A., Godoy S., Pereira L., Palma J., Rengifo M. (2006). Composición Fitoquímica y Nutricional de Algunos Frutos de Árboles de Interés Forrajero de Los Llanos Centrales de Venezuela. Rev. Cien. Fac. Vet..

[50] Ávalos-Soto J., Treviño-Neávez J.F., Verde-Star M.J., Rivas-Morales C., Oranday-Cárdenas A., Moran-Martinez J., Serrano-Gallardo L.B., Morales-Rubio M.E. (2014). Evaluación citotóxica de los extractos etanolicos de Azadirachta indica (A. Juss) sobre diferentes líneas celulares. Rev. Mex. Cienc. Farm..

[51] Apu A.S., Muhit M.A., Tareq S.M., Pathan A.H., Jamaluddin A.T.M., Ahmed M. (2010). Antimicrobial activity and brine shrimp lethality bioassay of the leaves extract of Dillenia indica Linn. J. Young Pharm..

